# Hyperpolarized ^13^C MRI Reveals Large Changes in Pyruvate Metabolism During Digestion in Snakes

**DOI:** 10.1002/mrm.29239

**Published:** 2022-04-15

**Authors:** Kasper Hansen, Esben Søvsø. S. Hansen, Nichlas Riise V. Jespersen, Hans Erik Bøtker, Michael Pedersen, Tobias Wang, Christoffer Laustsen

**Affiliations:** ^1^ Comparative Medicine Lab, Department of Clinical Medicine Aarhus University Aarhus Denmark; ^2^ Zoophysiology, Department of Biology Aarhus University Aarhus Denmark; ^3^ Department of Forensic Medicine Aarhus University Aarhus Denmark; ^4^ MR Research Centre, Department of Clinical Medicine Aarhus University Aarhus Denmark; ^5^ Cardiology, Department of Clinical Medicine Aarhus University Aarhus Denmark

**Keywords:** [1‐^13^C]pyruvate, metabolism, MRI, postprandial, python, reptile

## Abstract

**Purpose:**

Hyperpolarized ^13^C MRI is a powerful technique to study dynamic metabolic processes in vivo; but it has predominantly been used in mammals, mostly humans, pigs, and rodents.

**Methods:**

In the present study, we use this technique to characterize the metabolic fate of hyperpolarized [1‐^13^C]pyruvate in Burmese pythons (*Python bivittatus*), a large species of constricting snake that exhibits a four‐ to tenfold rise in metabolism and large growth of the visceral organs within 24–48 h of ingestion of their large meals.

**Results:**

We demonstrate a fivefold elevation of the whole‐body lactate‐to‐pyruvate ratio in digesting snakes, pointing to a large rise in lactate production from pyruvate. Consistent with the well‐known metabolic stimulation of digestion, measurements of mitochondrial respiration in hepatocytes in vitro indicate a marked postprandial upregulation of mitochondrial respiration. We observed that a low SNR of the hyperpolarized ^13^C produced metabolites in the python, and this lack of signal was possibly due to the low metabolism of reptiles compared with mammals, preventing quantification of alanine and bicarbonate production with the experimental setup used in this study. Spatial quantification of the [1‐^13^C]lactate was only possible in postprandial snakes (with high metabolism), where a statistically significant difference between the heart and liver was observed.

**Conclusion:**

We confirm the large postprandial rise in the wet mass of most visceral organs, except for the heart, and demonstrated that it is possible to image the [1‐^13^C]pyruvate uptake and intracellular conversion to [1‐^13^C]lactate in ectothermic animals.

## 1 INTRODUCTION

Hyperpolarized ^13^C MRI has emerged as a powerful experimental and clinical tool to study metabolic processes in vivo with the potential for early detection of metabolic changes in several diseases.^1^ Hyperpolarized ^13^C MRI has predominantly been used to study humans and mammalian model organisms such as mice,^2^, ^3^ rats^4^, ^5^ and pigs,^6^ although bacteria and protozoans have also been investigated.^7^, ^8^ The current study is, to the best of our knowledge, the first to study metabolism in a reptile with hyperpolarized ^13^C MRI. The most commonly used hyperpolarized substrate, [1‐^13^C]pyruvate, is a key central metabolic substrate for both glycolytic metabolism and oxidative phosphorylation, which are directly coupled to glucose homeostasis and thus influenced by the digestive state in an organ‐specific manner.^6^, ^9^, ^10^, ^11^, ^12^ Most mammals exhibit relatively modest metabolic responses to digestion compared to many nonmammalian vertebrates,^13^ and the fasted/fed transition has been extensively studied by hyperpolarized MR as an example of physiological adaptation that is quantifiable by the technique in health and metabolic disease.^12^, ^14^, ^15^, ^16^

Reptiles, such as pythons and other large constricting snakes, are adapted to endure months of fasting while retaining the capacity to digest very large meals that may amount to 20%–40% of their body mass.^17^, ^18^ This transition from famine to feast is associated with an impressive phenotypic flexibility for which the mass and function of many organs are upregulated within hours after ingestion.^13^, ^19^, ^20^, ^21^, ^22^, ^23^, ^24^, ^25^ Digestion in these animals is also accompanied by an extraordinary rise in metabolism in which the oxygen consumption rate increases more than fourfold for several days.^13^, ^24^, ^26^, ^27^ As a consequence, the Burmese python has been previously studied in order to understand extreme metabolic flexibility and potentially to provide insights to several human diseases.^28^, ^29^

The postprandial metabolic response in these animals appears entirely aerobic because plasma lactate levels remain low (< 0.5 mM^26^). Accordingly, the limited exchange of [1‐^13^C]pyruvate into the lactate pool represents a potential methodological limitation in reptiles because the exchange of ^13^C‐labeled pyruvate, catalyzed by lactate dehydrogenase (LDH), into this lactate pool is typically large in hyperpolarized experiments employed in mammalian species.^30^

Although hyperpolarized MRI increases the sensitivity of an injected tracer more than 10,000 times compared to conventional MRI,^31^ the inherently low metabolism in ectothermic vertebrates compared to endothermic mammals is a potential barrier to the use of hyperpolarized ^13^C in these animals. Therefore, this study aimed to evaluate the feasibility of hyperpolarized [1‐^13^C]pyruvate MRI in a nonmammalian ectothermic species, here represented by an ectothermic reptile, the Burmese python; and secondly to evaluate the well‐known and pronounced metabolic response to feeding after prolonged food deprivation in this species. This may well additionally function as an “upper bound” on the magnitude of physiological postprandial response that is expected to be detectable by the technique.

## 2 METHODS

All experiments were approved by the national animal experimental inspectorate (license: 2014‐15‐0201‐00100) and conducted following the Danish Ministry of Environment and Food Animal Research Act.

### 2.1 Animals, placement of a venous catheter, and anesthesia

Twelve Burmese pythons (*Python bivittatus*, Linnaeus 1758) were obtained from a commercial supplier and maintained individually in a dedicated reptile stable at 80% relative humidity in a 12/12 h day/night cycle. Because Burmese pythons are sensitive to variations in temperature and humidity, the scanning of fasted and fed animals was interleaved to ensure similar experimental conditions on the days of the scan. Room temperature was 28°C, and an electrical heat mat positioned at one end in each container provided a localized temperature of 32°C. At the time of the experiment, the snakes were 70 ± 2.5 months old. All snakes fasted for a minimum of 90 days while having free access to water before studies began. Six snakes (body mass: 1294 ± 52 g; snout‐cloaca length: 142 ± 2 cm) remained fasting during the studies, whereas the other 6 snakes (body mass: 1282 ± 38 g; snout‐cloaca length: 142 ± 2 cm) were allowed to ingest a large meal (∼25% body mass) approximately 48 h before the MRI examination. The reported body masses of the fed snakes were measured immediately prior to feeding.

To administer anesthetics and hyperpolarized pyruvate during MRI, a polyethylene catheter (PE 50) was inserted into the caudal vein, approximately 5 cm cranial to the cloaca, 50 h before MRI. To place the vascular catheter, anesthesia was induced by placing a bag containing isoflurane‐soaked gauze (IsoFlo Vet 100%, Abbot Laboratories, Berkshire, UK) over the head of the snake until reflexes subsided (within minutes). Then, the trachea was intubated for mechanical ventilation with 1%–3% isoflurane vaporized in oxygen (prepared by a Fluotec 3 Vaporizer, Simonsen & Weel, Albertslund, Denmark) using an anesthesia workstation (Halowell EMC, Pittsfield, MA). Body temperature was maintained at 30°C by a heat mat. Lidocaine (< 8 mg kg^−1^ in 1:1 saline) was injected subcutaneously to obtain local analgesia, and the catheter was inserted through a 3–5 cm incision. The entire procedure lasted no longer than 45 min, and all fed snakes ate voluntarily following the procedure.

**FIGURE 1:**
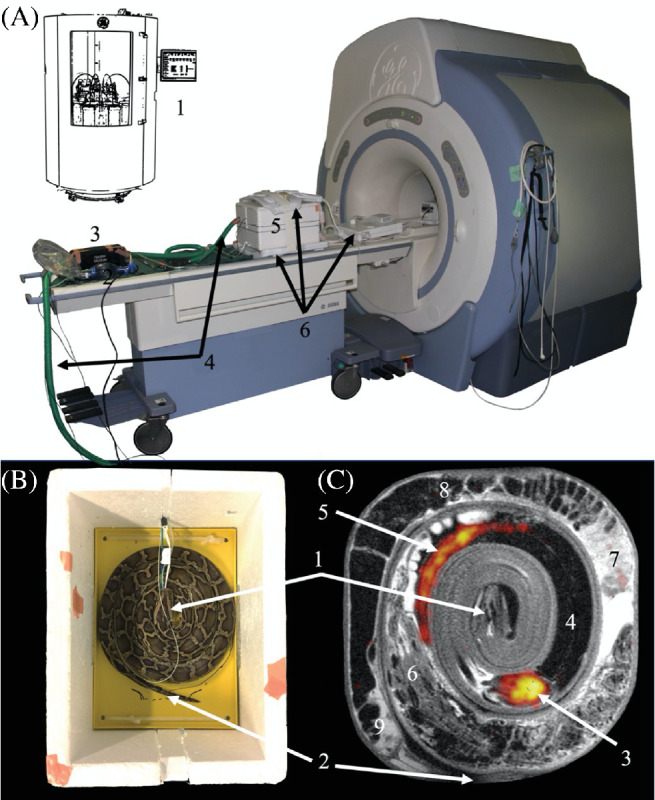
(A) Illustration of the experimental setup: (1) schematic of the dynamic nuclear polarization hyperpolarizer Spinlab (GE Healthcare, Brøndby, Denmark) (positioned in a separate room ∼5 m from MR system) used for hyperpolarization of [1‐^*13*^C]pyruvate; (2) neonate resuscitator bag, used for manual oxygen ventilation every fifth minute; (3) feedback‐controlled heating unit setup to maintain a body temperature of 30°C; (4) air hoses for the heating unit; (5) thermoregulated polystyrene box (with a python inside); and (6) surface coils for ^*1*^H MRI and ^*13*^C‐MRS. (B) The thermoregulated Styrofoam box containing 1 *Python bivittatus* (body mass: 1341 g, total length 165 cm). (C) Anatomical ^*1*^H image overlaid with a time‐averaged ^*13*^C image of [1‐^*13*^C]lactate. Annotation in (B, C): (1) head; (2) tail; (3) heart; (4) lungs; (5) liver; and the cranial‐to‐caudal position of the transition into the (6) stomach (containing undigested rats), (7) small intestine, and (8) colon, respectively, and (9) feces

Anesthesia to prevent spontaneous movement during imaging was provided by an intravascular injection of propofol (10 mg kg^−1^ propolipid, Fresenius Kabi, Sweden) and maintained with a propofol bolus (3 mg kg^−1^) every 20 min to induce anesthesia without having to handle the snakes—and hence avoiding the potential stimulation of anaerobic metabolism in connection with strenuous activity. The anesthetized snake was intubated and manually ventilated with oxygen using a neonate resuscitator bag every fifth minute during MRI. The snake was positioned in a spiral form in a custom‐fitted polystyrene box in which the temperature could be maintained at ∼30°C (Figure 1A, 1B).

Immediately after completing the MRI examination, the snakes were euthanized by cervical dislocation under propofol‐induced terminal general anesthesia. Following a rapid gross dissection, the visceral organs were weighed, and fresh liver samples were harvested for respirometry. Additional liver samples were obtained by a freeze clamp procedure to accommodate measurements of LDH activity, PDH activity, and lactate concentration.

### 2.2 MRI

**FIGURE 2:**
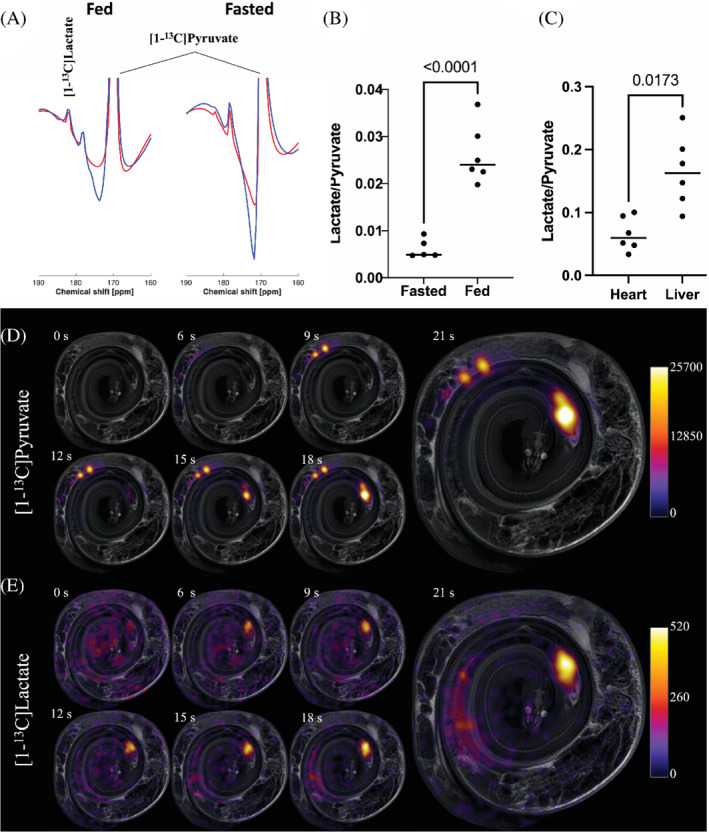
(A) Summed [1‐^13^C]pyruvate spectra (blue) and AMARES fitted result (red) ([1‐^13^C]pyruvate: 171 ppm), showing a substantial [1‐^13^C]lactate peak (at 183 ppm) in postprandial animals with a 183% increased absolute lactate signal compared to the fasted animals (left), whereas the [1‐^13^C]lactate peak from fasted animals (right) is almost absent. (B) Plot of the lactate‐to‐pyruvate ratio for all snakes, showing a statistically significant increased lactate production in postprandial (fed) animals (*P* < 0.0001). (C) Plot showing a statistical significantly increased lactate production in the liver, compared to the heart of postprandial animals (*P* = 0.003). (D, E) Time series of [1‐^13^C]pyruvate (D) and [1‐^13^C]lactate (E) and at 0, 6, 9, 12, 15, 18, and 21 s after administration of 4 mL 125 mM hyperpolarized [1‐^13^C]pyruvate in 1 postprandial python (legends display raw spectral MR‐signal). [1‐^13^C]lactate (D) and [1‐^13^C]pyruvate (E) images are overlaying anatomical ^1^H images (for annotation compare with the animal in Figure 1C). These time series show that [1‐^13^C]pyruvate was distributed in the whole body, with hotspots in the heart and the proximal part of the colon, and that [1‐^13^C]lactate was accumulated mainly in the liver and heart (E) over the 21 s after administration of the [1‐^13^C]pyruvate‐tracer

**FIGURE 3:**
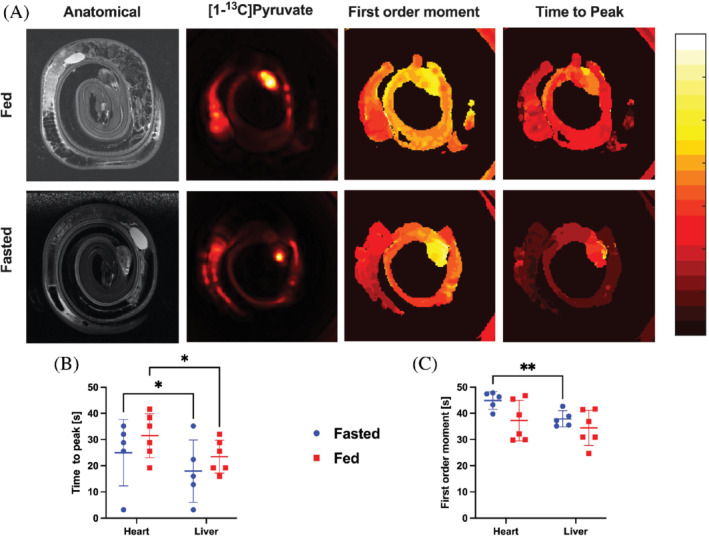
(A) From left to right: Anatomical images, the summed [1‐^13^C]pyruvate distributional patterns in % signal to maximum signal, first‐order moments (FM) [0–70] in seconds, and TTP [0–70] in seconds were similar in in the fed (top) and fasted (bottom) animals. (B) TTP regional analysis of the heart and liver revealed a statistically significant difference between the organs (*P* = 0.002) but no response to feeding (*P* = 0.32), indicating no significant perfusion difference with feeding status. Also, no interaction was observed (organ × feeding, *P* = 0.79). (C) First‐order moment analysis of the heart and liver revealed a statistically significant difference between the organs (*P* = 0.004), whereas neither the response to feeding *P* = 0.12 nor an interaction were significant (*P* = 0.13, organ × feeding). An asterisk denotes interorgan statistical significance. *N* = 5 fasted and *N* = 6 fed animals. TTP, time‐to‐peak

The animals were imaged with a 3 Tesla GE HDx MRI system (GE Healthcare, Milwaukee, WI, USA). ^1^H images were acquired with an 8‐channel cardiac array receiver coil (GE Healthcare, Milwaukee, WI, USA) and ^13^C images with a 20 cm diameter Helmholtz loop coil (PulseTeq Limited, Surrey, UK). [1‐^13^C]pyruvate was polarized using a Spinlab (GE Healthcare, Brøndby, Denmark) as previously described.^4^ Each animal was injected with 4 mL (125 mM) hyperpolarized [1‐^13^C]‐pyruvate. During MRI the snakes were positioned in a spiral shape in the ^13^C coil (Figures 1B, C, 2D–E, 3B). A fixed distance between the coils was obtained by using a customized polystyrene box, which, as noted above, also served as an acclimatization chamber to maintain a setpoint temperature of 30°C (Figure 1A). Temperature regulation was accomplished with an MRI‐compatible monitor for veterinary use (Model 1035, SA Instruments, Stony Brook, NY), which was feedback‐controlled from internal body temperature readings in the cloaca. Images were acquired in the coronal plane for whole animal coverages (Figures 1C, 2D–E, 3A).

For anatomical visualization, a T_2−_weighted [^1^H] fast spin echo sequence was acquired using the following parameters: TE/TR = 108 ms/2500 ms; averages = 3; flip angle = 90°; FOV = 320 × 320 mm^2^; slice thickness = 2 mm; and image resolution = 1.1 × 1.1 mm^2^. For mapping of the pyruvate metabolism, a 128 s continuous data acquisition was initiated simultaneously with the beginning of the 4.0 mL bolus injection of [1‐^13^C]pyruvate into the caudal vein. ^13^C images were acquired using a ^13^C iterative decomposition of water and fat with echo asymmetry and least squares estimation (IDEAL) multi‐echo spiral scan with the following parameters: acquisition rate per image = 3.2 s; 11 echoes and 1 initial spectrum per IDEAL encoding; TR/TE/∆TE = 100 ms/1.1 ms/0.9 ms; flip angle = 10°; FOV = 200 × 200 mm^2^; slice thickness = 50 mm covering the complete animal; and image resolution: 2.5 × 2.5 mm^2^. The reconstruction of the multi‐echo spiral acquisition was performed with a standard [1‐^13^C]pyruvate IDEAL reconstruction^32^ allowing for differentiation of pyruvate, alanine, pyruvate hydrate, lactate, and bicarbonate.

Region of interest analyses of anatomical T_2_‐weighted images were performed using Osirix image analysis software (Pixmeo, Geneva, Switzerland). Maps of pyruvate and lactate were adjusted to the maximum signal levels and were then overlaid on the acquired anatomical T_2_‐weighted images (Figures 1C, 2D, E). Regions of interest were drawn on the heart and liver on the anatomical ^1^H images and transferred to the ^13^C images to extract the spatially encoded hyperpolarized data. The whole animal metabolic conversion was performed by fitting the individual spectra of the complete slab using the OXford Spectroscopy Analysis (OXSA) advanced method for accurate, robust, and efficient spectral fitting (AMARES) fitting routine^33^ (Supporting Information Figure S2). The area‐under‐the‐curve ratio‐metric approach was used as a measure of metabolic conversion from [1‐^13^C]pyruvate to [1‐^13^C]lactate.^34^ Only images originating from the fed state were included in the spatial image analysis (heart and liver) due to a low SNR observed in the interleaved spectroscopy analysis in the fasted pythons. Pyruvate hemodynamics was evaluated using the raw signal (Supporting Information Figure S1), time‐to‐peak, and first‐order moment of the pyruvate signal.

### 2.3 Mitochondrial purification

Liver samples were homogenized in a Dounce tissue homogenizer (5 strokes rotating 2–300 rpm) in an isolation medium (250 mM sucrose, 10 mM HEPES, 1 mg mL^−1^ bovine serum albumin fatty acid‐free, pH 7.4, 300 mOsm kg^−1^ H_2_O). The homogenate was centrifuged (800×*g* for 10 min) to remove tissue debris in the pellet and lipid layers on the surface. This homogenate was then further centrifuged (14 500×*g* for 5 min). Dead mitochondria were rinsed away, and the pellet (intact mitochondria) was resuspended in Bovine serum albumin‐free isolation medium. The homogenate was then centrifuged again (14 500×*g* for 5 min) and resuspended in a preservation medium (250 mM sucrose, 5 mM HEPES, pH 7.4, 300 mOsm kg^−1^ H_2_O).

### 2.4 Mitochondrial respiratory capacity

Mitochondrial respiratory oxygen consumption was recorded using a high‐resolution respirometry (Oxygraph‐2k; Oroboros Instruments, Innsbruck, Austria). The oxygen consumption was normalized to the number of intact mitochondria using the mitochrondial enzyme fumarase activity. Mitochondrial function was determined as state 2 respiration in the presence of complex I‐linked substrate glutamate (10 mM) and malate (2 mM). State 3 respiration was determined after the addition of adenosine diphosphate (5 mM) including glutamate and malate. State 3S was determined after the addition of complex II‐linked substrate succinate (10 mM), including adenosine diphosphate. State 4, or leak respiration, was determined in the presence of the adenosine triphosphate‐synthase inhibitor oligomycin A (2 μg mL^−1^). Uncoupled respiration was determined after the addition of carbonyl cyanide‐p‐trifluoromethoxyphenylhydrazone (1.5–2 μM). Nonmitochondrial respiration was measured in the presence of the complex I‐inhibitor rotenone (0.5 μM) and the complex III‐inhibitor antimycin A (2.5 μM). The respiratory control ratio was calculated as state 3/state 2.

### 2.5 Hepatic enzyme activity

LDH and PDH activity and lactate concentration assays were performed according to the manufacturer's instructions (Sigma Aldrich, Brøndby, Denmark) with few alterations. Tissue was homogenized in phosphate‐buffered saline buffer for 50 s at 20 000 Hz by a Bandelin Sonoplus ultrasonic homogenizer (Buch & Holm, Herlev, Denmark) and then centrifuged at 3500 rpm at 22°C for 10 min. The total protein concentration of the homogenate was measured using a Pierce BCA protein assay kit (Thermo Scientific, Roskilde, Denmark). Analysis was performed on either 96 or 384 well plates in a BioTEK synergy plate reader (AH diagnostics, Aarhus, Denmark). Activity measurements of tissue were normalized to protein content in the sample solution.

### 2.6 Statistics

The difference in mean values between fasted and fed groups was compared using a Student's *t*‐test for parametric data and Wilcoxon's rank‐sum test for nonparametric data. Statistical analysis of basic animal and dissection data was made using R (RStudio, Version 1.1.456), whereas statistical analysis of hyperpolarized data, enzyme activity, and mitochondrial respiration was performed using GraphPad Prism (version 9). The hemodynamic responses were analyzed using a 2‐way repeated analysis of variance (taking into account that the liver and heart were from the same animals.

## 3 RESULTS

Body mass and length of fasted and digesting snakes were similar (*P* = 0.86 and *P* = 0.9, respectively). Due to technical problems with the 1 of 12 snakes was not injected with [1‐^13^C]pyruvate. Consequently, we report complete hyperpolarized data from 5 fasted and 6 digesting snakes. Organ mass and liver mitochondrial respiration as well as LDH and PDH‐activities and lactate concentration were assessed in all 12 snakes (*n* = 6 in each group). The cloacal temperature was stable during the MRI scans in both fasting and digesting snakes (30.0 ± 0.1°C and 30.6 ± 0.3°C, respectively; average ± standard error of the mean).

### 3.1 Phenotypic flexibility of visceral organs

The ingestion of a large meal following prolonged fasting elicited a statistically significant increase in the mass of liver, stomach, pancreas, small intestine, both kidneys, and the total mass of right and left atria (Table 1). There was no statistically significant change in the mass of the 1‐chambered cardiac ventricle (*P* = 0.1). The gall bladder mass of postprandial animals was reduced compared to fasted animals, but the difference was not statistically significant (*P* = 0.3) (Table 1).

**TABLE 1:** Dissection

	Fasted	Fed	*T* Test	Difference (%)
	(g or g kg^−1^ ± SEM [*n*])	(g or g kg^−1^ ± SEM [*n*])	*P* value	(fed vs. fasting)
Length (cm) (snout: cloaca)	141.8 ± 1.8 (*n* = 6)	142.2 ± 2.1 (*n* = 6)	*P* = 0.905	0.2
Body mass (g)	1294.0 ± 52.1 (*n* = 6)	1282.0 ± 38.0 (*n* = 6)	*P* = 0.856	−0.9
Liver (g kg^−1^)	11.042 ± 0.38 (*n* = 5)	21.07 ± 2.06 (*n* = 6)	*P* < 0.001	90.8*
Stomach (g kg^−1^)	17.978 ± 1.21 (*n* = 5)	27.12 ± 1.25 (*n* = 6)	*P* < 0.001	50.8*
Pancreas (g kg^−1^)	0.886 ± 0.048 (*n* = 5)	1.33 ± 0.12 (*n* = 6)	*P* = 0.007	50.1*
Gallbladder (g kg^−1^)	10.59 ± 1.01 (*n* = 4)	9.232 ± 0.75 (*n* = 5)	*P* = 0.32	−12.8
Small intestine (g kg^−1^)	18.238 ± 1.88 (*n* = 6)	30.496 ± 0.918 (*n* = 6)	*P* < 0.001	67.2*
Kidney, left (g kg^−1^)	2.368 ± 0.197 (*n* = 6)	3.808 ± 0.187 (*n* = 6)	*P* < 0.001	60.8*
Kidney, right (g kg^−1^)	2.345 ± 0.12 (*n* = 6)	3.978 ± 0.266 (*n* = 6)	*P* < 0.001	69.7*
Atrial mass (sum of left and right) (g kg^−1^)	0.433 ± 0.035 (*n* = 6)	0.602 ± 0.055 (*n* = 5)	*P* = 0.036	38.9*
Ventricle mass (g kg^−1^)	1.965 ± 0.074 (*n* = 6)	2.436 ± 0.2415 (*n* = 5)	*P* = 0.124	24.0

Abbreviation: SEM, standard error of the mean.

* An asterisk denotes a significant difference between fasted and fed animals; *P* < 0.05.

Length from snout to cloaca, absolute body mass, and body mass normalized masses of liver, stomach, pancreas, gallbladder, small intestine, left and right kidneys, the sum of left and right atria, and the (anatomically undivided) ventricle from fasted (90 days) and fed pythons 48 hours after receiving a full rodent meal of approximately 25%/body mass (in the fed animals, body mass was measured immediately prior to feeding).

### 3.2 Metabolic flexibility

We observed a dramatic, statistically significant mean increase of 421% in the lactate‐to‐pyruvate ratio from the fasted to the fed animals (*P* < 0.0001), indicating the production of lactate from pyruvate (Figure 2E). It was not possible to quantify the amino acid production via alanine measurement nor the aerobic metabolism via the tricarboxylic acid cycle as potentially verified by the production of HCO_3_^−^: these metabolites, readily seen in mammals, were not detectable in either state. Only in the postprandial state, where the SNR was found to be high enough, were we able to obtain a reliable spatial quantification with IDEAL. We found a statistically significant intrapython interorgan difference between the heart and liver (*P* = 0.017) (Figure 2).

### 3.3 [1‐^13^C]Pyruvate hemodynamics

To overcome this limitation in the data, we analyzed the high SNR [1‐^13^C]pyruvate dynamics images using time‐to‐peak as a surrogate metric for perfusion. We found no statistical significant difference in time‐to‐peak between the fasted and fed animals (*P* = 0.32), whereas a statistically significant shorter pyruvate delivery time was seen in the liver in contrast to the heart (*P* = 0.002) (Figure 3A,B). No statistical significant interaction was seen (*P* = 0.79, organ × feeding) for the pyruvate delivery (Figure 3B). The first‐order moment (FM) can be regarded as the time point when a substrate is retained in a given tissue and thus is directly linked to the metabolic conversion as well as the perfusion of the tissue. We found a 12% shorter FM in the liver compared to the heart (*P* = 0.002), whereas no statistical difference was seen between the 2 feeding states (*P* = 0.12), or any interaction between feeding and the organ imaged (*P* = 0.13, organ × feeding) (Figure 3C).

### 3.4 Hepatic mitochondrial respiration

Overall mitochondrial oxygen consumption rates were significantly higher in fed in comparison with fasting pythons (Table 2). A statistically significant increase in state 1–3 respiration was also observed between fasted and fed animals, whereas state 4 respiration remained unchanged. The respiratory control ratio was statistically significantly higher in fed compared to fasted animals, indicating an increased capacity for aerobic metabolism. Leak respiration was higher in postprandial compared to fasted animals, which suggested efficient utilization of the higher ATP production in the postprandial animals (Table 2).

**TABLE 2:** Hepatic Mitochondrial Respiration

Mitochondrial States	Fasted (pmolO_2_ s^−1^*mg)	Fed (pmolO_2_ s^−1^*mg)	Difference (%)	*P* Value
State 2	44.3 ± 3.6	96.9 ± 11.4	118.6	*P* = 0.034*
State 3	132.6 ± 53.3	1840.7 ± 273.4	1288.5	*P* = 0.021*
State 3S	459.2 ± 174.4	2448.7 ± 360.8	433.3	*P* = 0.017*
State 4: Oligomycin	212.2 ± 26.2	277.3 ± 29.6	30.6	*P* = 0.168
RCR	3.2 ± 1.5	18.9 ± 0.6	487.3	*P* = 0.0009*
Uncoupling FCCP	1078.8 ± 134.7	1751.3 ± 148.2	62.3	*P* = 0.023*

Abbreviations: FCCP, trifluoromethoxyphenylhydrazone; RCR, respiratory control ratio.

* An asterisk denotes a significant difference between fasted and fed animals; *P* < 0.05.

The applied feeding after fasting protocol elicited significant up‐regulation of several states. During state 2; state 3 (complex I‐linked); state 3 s (complex I and II‐linked); and state 4 with oligomycin, RCR, and uncoupling with FCCP. All values are group mean ± SEM.

### 3.5 Hepatic enzyme activities

A statistically insignificant numerical decrease between fasted to fed animals in hepatic LDH activity (from 38 to 33 mU mg_protein_^−1^, respectively) and lactate concentration (from 0.98 to 0.91 mM, respectively), and an increase in PDH activity (from 0.098 to 0.11 mU mg_protein_^−1^, respectively) were found in measurements from liver samples (Supporting Information Figure S2).

## 4 DISCUSSION

We have demonstrated the feasibility of hyperpolarized [1‐^13^C]pyruvate MRI for measuring the in vivo metabolism of an ectothermic nonmammalian vertebrate, and we report statistically significant postprandial changes in metabolism, including a fourfold rise in whole‐body lactate production during digestion, representing one of the highest increases in metabolism recorded by the technique. The upregulation of lactate production in postprandial snakes was sufficient to image the organ distribution, particularly in the liver, and to some extent also in the heart (Figure 2E). However, the received signal from alanine and bicarbonate were not sufficient to enable an accurate quantification using the given setup. This lack of signal relates either to the low overall metabolism of ectothermic vertebrates compared with mammals, a decrease in the presence of alanine transferase or flux through pyruvate dehydrogenase, or to the relatively low tissue perfusion in ectothermic animals. However, because the pyruvate images showed a clear distribution in the entire animal (Figures 2D and 3B), we believe that the low metabolism in this species is probably a more likely cause of the lacking signal from alanine and bicarbonate than low blood perfusion. This is supported by the time‐to‐peak and FM analysis methods, which are both long (23–31 s and 36–41 s, respectively) compared with mammals, where they are on the order of a few seconds to 20 s, respectively, in similar sized animals^35^, ^36^: differences in heart rate alone are not sufficient to explain this because the pyruvate bolus will be well mixed within a minute. A high metabolic conversion would reduce the pyruvate retention in the tissue and thus FM, whereas reduced perfusion would increase the FM.

Interestingly, our data also show a significantly higher mean absolute pyruvate signal and variation in fasted compared to fed animals. Pythons have a significantly reduced whole blood volume in the postprandial state (16.5 ± 1.7 mL kg^−1^ vs. 9.7 ± 1.0 mL kg^−1^ fasted/fed mean ± standard error of mean^37^). This complicates the interpretation of our study: the bolus of the tracer injected was fixed in this experiment, meaning that fasted pythons may have had a lower total effective concentration of pyruvate in their blood, which could potentially explain the larger pyruvate area under the curve variation and why the downstream metabolites are not observable in the fasted state. A potential explanation for the lower absolute signal in the fed group might be increased mitochondrial uncoupling for heat production,^38^, ^39^ an increased uptake, and subsequent relaxation of pyruvate in tissue due to the observed increase in visceral organ mass.

The higher metabolism in the fed state is supported by the absolute increased lactate SNR (Supporting Information Figure S1) and the fact that the relative FM difference is much smaller than the difference between the absolute pyruvate signal. Further studies are needed to address this surprising finding. An increased uptake and generally upregulated metabolism is supported by similar maximum capacity for LDH and PDH activity between the 2 feeding states (Supporting Information Figure S2).

The significant rise in the visceral organ mass during digestion is consistent with a number of previous reports.^18^, ^20^, ^22^, ^40^, ^41^, ^42^, ^43^ Also, in line with earlier studies, we show that the cardiac ventricle does not grow in digestion^42^, ^43^ and any cardiac hypertrophy is driven by filling pressures and hemodynamic effects.^37^

It has been previously demonstrated that the rate of oxygen uptake increases up to 5 times when Burmese pythons digest meals that correspond to 20%–25% of their body mass in the absence of any rise in circulating plasma lactate levels (< 0.5 mM).^26^, ^27^ In hepatic samples from both fasted and fed animals, we measured comparable lactate concentrations of ∼0.94 mM (Supporting Information data Figure S2) in both cases. Although ^13^C‐bicarbonate was most likely below the limit of detection in our hyperpolarized [1‐^13^C]pyruvate experimental setup, it is probably that the upregulation of anaerobic pathways found in this study was accompanied by an equivalent rise in pathways for pyruvate oxidation.^13^, ^24^, ^26^, ^27^

For the Burmese python, the digestion of meals such as those employed in the present study (25% of body mass) is a slow process that lasts for up to a week.^22^ Here, pythons were studied at 48 h into their postprandial period because it has been documented that the metabolic response to digestion in this species peaks around this time point,^18^, ^26^, ^27^ which is supported by the finding that most of the organ growth and remodeling has occurred at this stage.^18^, ^21^, ^22^, ^24^ We therefore argue that this study demonstrates a novel physiological “maximal increase” of detectable glycolytic metabolism as measured by hyperpolarized [1‐^13^C]pyruvate.

Our measurements on liver mitochondria revealed marked upregulation of mitochondrial respiration in the postprandial animals that may support a concomitant several‐fold rise in oxidative metabolism. Previous studies have demonstrated increased oxidative mitochondrial capacity during digestion in other vertebrates, including penguins and fish,^44^, ^45^, ^46^, ^47^ but not with the order of magnitude reported here. In a parallel unpublished study on smaller *Python bivittatus*, we found no digestion‐induced changes in mitochondrial respiration on permeabilized cells from the cardiac ventricle, liver, and mucosa from the stomach and small intestine after 8 weeks of fasting. Given that the snakes had fasted for more than 13 weeks in the present study, it is likely that the downregulation of mitochondrial respiration takes place in prolonged food deprivation, where reductions in metabolism will extend survival on bodily energy reserves.^13^ The regulation of mitochondrial function during the transition from fasting to digestion is an interesting topic for further studies, particularly the investigation of the underlying mechanisms and signaling for the transition between metabolic profiles. The hyperpolarized pyruvate and downstream metabolite signals represent the effective metabolic uptake and conversion of pyruvate and thus are limited by the expression of monocarboxylate transporters, metabolite and co‐substrate pool sizes, and enzyme concentrations and their phosphorylation. Accordingly, in ectothermic animals the influence of these underlying genomic and metabolomic features deserves further investigation.

## 5 CONCLUSION

Overall, our findings support a general upregulation of both the aerobic and anaerobic downstream metabolic pathways of pyruvate in postprandial compared to fasting pythons. An explanation for the metabolic difference between the heart and liver might be the shift away from hepatic gluconeogenesis, as indicated by a buildup of intracellular lactate in the liver,^5^, ^48^, ^49^ which was not found in the myocardium. Reptiles, however, rely less on hepatic gluconeogenesis than do mammals after strenuous exercise,^50^ and whether a similar difference applies for digestion remains to be studied.

Several factors limit the scope of this feasibility study, including both hardware and choice of the pulse sequence. Improved RF coil performance, array coils, and a closer position of the coil to the animals as well as spectral–spatial excitation schemes are believed to improve the SNR and thus potentially the quantification of downstream metabolites.^51^, ^52^, ^53^, ^54^ Furthermore, variation in the circulating blood volume of the python may have hindered our ability to quantify metabolism in the fasted state: future studies may move from a constant bolus dose scheme to a dose titrated to an individual's estimated (but unknown) blood volume.

This study demonstrated that hyperpolarized MRI is a feasible technique for obtaining spatial and temporal information about the metabolism of selected biomolecules fundamental to biological function in ectothermic animals in vivo.

## Supporting information

FIGURE S1. Left. The mean [1‐^13^C]pyruvate time curve from fasted (*N* = 5) (black) and fed (*N* = 6) (gray) pythons. Dashed lines indicate the standard deviation. Right. Area under the curve (AUC) of the absolute signal level of the pyruvate curve.
FIGURE S2. There were no statistically significant differences in hepatic LDH (*P* = 0.67) and PDH activity (*P* = 0.12), nor in lactate concentration (*P* = 0.44) between the fasted and fed pythons.
