# Supplementary material for: Hyperpolarized 13C MRI Reveals Large Changes in Pyruvate Metabolism During Digestion in Snakes
Source: Magn Reson Med. 2022 Apr 15;88(2):890–900. doi: 10.1002/mrm.29239 (PMC9321735; doi:10.1002/mrm.29239)
Supplement: Supplementary file 1 — FIGURE S1. Left. The mean [1‐13C]pyruvate time curve from fasted (N = 5) (black) and fed (N = 6) (gray) pythons. Dashed lines indicate the standard deviation. Right. Area under the curve (AUC) of the absolute signal level of the pyruvate curve. FIGURE S2. There were no statistically significant differences in hepatic LDH (P = 0.67) and PDH activity (P = 0.12), nor in lactate concentration (P = 0.44) between the fasted and fed pythons. [file MRM-88-890-s001.docx]

[1-^13^C]*Pyruvate signal: hemodynamic and absolute levels*

The hyperpolarized [1-^13^C]pyruvate signal was found to be statistically significantly higher in the fasted compared with fed pythons (p = 0.0022) (Figure 1). This result supports the overall conclusion that higher uptake and metabolic conversion of pyruvate is present in the fed compared with fasted pythons. A relative larger variation is observed in the fasted animal compared to the fed animals; this is likely biological variations as the examination was consistent between groups. This indicate that it is beneficial to image the animals in the fed state as it lowers the variation.

**Absolute [1-^13^C]pyruvate signal dynamics**

**Supporting Information Figure S1**. Left. The mean [1-^13^C]pyruvate time curve from fasted (black) and fed (gray) pythons. Dashed lines indicate the standard deviation. Right. Area under the curve (AUC) of the absolute signal level of the pyruvate curve.

*Fitting procedure*

Pyruvate and lactate signals were estimated using the AMARES fitting routine (1). Although other metabolites (pyruvate, pyruvate-hydrate, lactate, alanine and bicarbonate) were used as prior-knowledge in the model fit, only lactate could be accurately quantified (Figure 2), as the signal to noise (SNR) level for other metabolites was below the detection/quantification level of the experimental setup (see figure 2).

*Hepatic enzyme activity results*

Hepatic LDH (p = 0.67) and PDH activity (p = 0.12) as well as lactate concentration (p = 0.44) were found to be largely similar between the fasted and fed animals, with a numerical but non-significant decrease in LDH activity and lactate concentration, and an increase in PDH activity (Figure 3).

**Biochemical assays of hepatic enzyme activities and lactate concentrations**

**Supporting Information Figure S2**. There were no statistically significant differences in hepatic LDH (p = 0.67) and PDH activity (p = 0.12), nor in lactate concentration (p = 0.44) between the fasted and fed pythons.

**References**

1. Purvis LAB, Clarke WT, Biasiolli L, Valkovič L, Robson MD, Rodgers CT. OXSA: An open-source magnetic resonance spectroscopy analysis toolbox in MATLAB. PLOS ONE 2017;12:e0185356 doi: 10.1371/journal.pone.0185356.
